# Inter-rater agreement of an alternative guideline for the assessment of the activities of daily living in home nursing

**DOI:** 10.1186/2049-3258-73-S1-P44

**Published:** 2015-09-17

**Authors:** Louis Paquay, Lut De Prins, Kelly Wauters, Kristel De Vliegher

**Affiliations:** 1 Wit-Gele Kruis Vlaanderen, Brussels, Belgium; 2 Rijksinstituut voor ziekte en invaliditeitsverzekering, Brussels, Belgium

## Aim

To evaluate the effect of an alternative guideline on inter-rater agreement of assessments of the activities of daily living (ADL) performed by home care nurses and advising nurses of health insurance organisations.

## Background

High accuracy of assessments of ADL dependency is needed for correct reimbursement of nursing care. Health insurance organisations often report high numbers of disagreement between home care nurses and advising nurses on the assessments of functional status in ADL of patients receiving home nursing.

## Method

A quantitative comparative field study in a stratified random sample of 612 patients receiving home nursing for ADL deficiencies. A proportional stratification according to mutuality membership, region (Flanders, Brussels and the Walloon region) and the patient's level of ADL-dependency in 5 categories: minor dependency (T2 en T7), level A, level B, level C (which is the highest level) was performed. Home care nurses were invited by the ‘National College of Advising Physicians (NCAPh) and the Belgian sickness funds, to participate in the study and to perform ADL assessments according to two guidelines: the current guideline of the Belgian Evaluation Scale (BES) and an alternative guideline which was written by a workgroup of the Agreement Commission for home care nursing of the National Institute for Health and Disability Insurance. The weighted kappa statistic (κ) (and its 95% confidence interval) and the proportion agreement were the main outcome measures.

## Findings

Inter-rater agreement for BES total score was κ = 0.70 (0.66 - 0.74) and 63.7% proportion agreement for the current guideline; κ = 0.60 (0.55 - 0.64) and 53.8% agreement for the alternative guideline.

## Conclusion

Inter-rater agreement of assessments using the current guideline was substantial and did not improve using the alternative guideline. There was no evidence for adopting the alternative guideline in legislation.

